# Gating pain; from normal to pathological sensory coding

**DOI:** 10.1080/24740527.2017.1329295

**Published:** 2017-05-22

**Authors:** Yves De Koninck

**Affiliations:** Laval University, Quebec Mental Health Institute, Quebec, Canada

A key to the future of chronic pain management is to understand the neurobiological mechanisms that govern how our brain adapts and maladapts to an imbalance in our sensory system following an injury to our body or a disease condition. This is critical to be able to target the root cause of abnormal, pathological pain for therapeutics. The current drama is that most drugs used for treating chronic pain to date have emerged from off-label use and are therefore not designed to directly address the source the problem. I present work my trainees and I have pursued to identify key mechanisms explaining pathological pain as well as co-morbidities associated with prolonged pain hypersensitivity. This includes the discovery of impaired inhibition resulting from chloride dysregulation in neuropathic pain leading to cross-talk between sensory channels (allodynia), possibly underlying spontaneous pain. I will illustrate how such discovery open new perspectives to understand abnormal pain and how it affects our thinking for therapeutic design. I will also describe how this work has led us to unravel some basic mechanisms underlying the adaptive and maladaptive response to opiate treatment, revealing that opiate tolerance, hyperalgesia and withdrawal result from distinct mechanisms. Each can be targeted independently, without affecting the analgesic effect of opiates, introducing avenues for adjuvant therapies to improve prolonged opiate use. I will conclude with some outlooks on future prospects for pain research, exploiting light to probe and manipulate pain micro-circuits, linking cellular and molecular studies to behavior, an essential step towards improving translation of basic research findings into clinical applications.

